# A human-scale porcine fasciocutaneous and muscle flap model for the evaluation of ortho-plastic reconstructions of lower-limb defects

**DOI:** 10.5194/jbji-10-597-2025

**Published:** 2025-12-10

**Authors:** Josefine Slater, Maiken Stilling, Andreas Engel Krag, Sara Kousgaard Tøstesen, Mads Kristian Duborg Mikkelsen, Martin McNally, Alexander James Ramsden, Louise Kruse Jensen, Birgitte Jul Kiil, Mats Bue

**Affiliations:** 1 Aarhus Denmark Microdialysis Research (ADMIRE), Orthopaedic Research Laboratory, Aarhus University Hospital, Aarhus, 8200, Denmark; 2 Department of Clinical Medicine, Aarhus University, Aarhus, 8200, Denmark; 3 Department of Orthopaedic Surgery, Aarhus University Hospital, Aarhus, 8200, Denmark; 4 Department of Plastic and Breast Surgery, Aarhus University Hospital, Aarhus, 8200, Denmark; 5 Bone Infection Unit, Nuffield Orthopaedic Centre, Oxford University Hospital, Oxford, OX3 7LD, United Kingdom; 6 Department of Veterinary Animal Sciences, University of Copenhagen, Frederiksberg, Denmark

## Abstract

**Introduction**: Fasciocutaneous and muscle flaps are used for the reconstruction of lower-limb composite bone and soft-tissue defects. Flap-mediated contributions to the healing microenvironment are less described. We present a comparative porcine model with a standardized bone and soft-tissue defect reconstructed by fasciocutaneous or muscle flaps and characterize the early tissue response following flap transfer. **Methods**: Using both hindlimbs of 10 female pigs, symmetrical tibial bone and soft-tissue defects were created and reconstructed with fasciocutaneous flaps ( n= 8) or muscle flaps ( n= 8) or were closed primarily ( n= 4, controls). Interstitial metabolites (glucose, lactate, and pyruvate) were sampled by microdialysis from flap and control tissue for 11 h before, during, and after 60 min of global flap ischemia (simulating free flap transfer). Flap histopathology was graded for the acute inflammatory response. **Results**: All pigs and flaps completed the study. Both flaps exhibited ischemia–reperfusion-induced metabolic alterations relative to the control tissue. The lactate-to-pyruvate ratio increased 3-fold in muscle flaps during ischemia, while fasciocutaneous flaps showed lactate and a lactate-to-pyruvate ratio that were 1.5-fold higher during reperfusion. Histopathology demonstrated early cellular activity at the bone lesion–flap interface in both flap types, with greater oedema and hyperaemia in fasciocutaneous flaps. **Conclusion**: We established a reproducible comparative large-animal model integrating interstitial metabolite and histopathological analyses to describe early flap-mediated tissue responses. Early flap-specific differences in metabolic and structural patterns may influence flap function and the healing microenvironment. The model provides a basis for evaluating clinically relevant ortho-plastic outcomes.

## 1 Introduction

Extensive trauma, tumour resection, or infection affecting the lower limb frequently require ortho-plastic reconstructive surgery for limb salvaging. Soft-tissue defects of the distal third of the limb are particularly challenging and usually necessitate free tissue transfer for reconstruction (Kozusko et al., 2019; Marais et al., 2024; Angelini et al., 2024). Yet, no controlled, randomized study has been conducted to demonstrate the superiority of one flap type over another. Current clinical practise renders flap choice dependent on many factors, including defect size, depth, need for composite tissues (e.g. bone, tendon, nerve supply), and contour requirements.

Defects in the lower limb often present as a composite of bone and soft tissues, creating a poorly vascularized tissue void described as a dead space (Cierny et al., 2003; Metsemakers et al., 2020). This tissue microenvironment has a significant effect on wound, soft-tissue, and bone healing, mainly by altering tissue oxygenation, nutrient supply, immune cell activity, growth factors, and cytokine signalling (Lu et al., 2007; Short et al., 2022; Schultz et al., 2011). Furthermore, a compromised microenvironment can facilitate bacterial growth (Bjarnsholt et al., 2022; Lu et al., 2007). Previous animal studies have demonstrated that complete re-vascularization of injured bone may take up to 4 weeks (Fisher and Wood, 1987; Lu et al., 2007). Early reconstruction of complex defects with well-vascularized soft tissue to fill the dead space is therefore crucial to restore perfusion and enable antibiotic delivery for tissue healing and infection control. Although fasciocutaneous tissues have been associated with higher vascular densities, muscle tissues have demonstrated superior bone healing and bacterial suppression (Harry et al., 2009, 2008; Richards et al., 1991; Gosain et al., 1990).

Beyond the technical and functional aspects of ortho-plastic flap selection, the flap's capacity to establish a microenvironment that promotes healing and infection control should also be considered. Hence, we developed a comparative large-animal model with a standardized composite bone and soft-tissue defect, the soft tissues of which were reconstructed by either a muscle or a fasciocutaneous flap. The primary objective of this study was to substantiate the model by characterizing the early tissue response following flap transfer through (1) interstitial metabolite profiles assessed by microdialysis and (2) histopathological analysis of the acute inflammatory response. This model provides a future platform for investigating the biological basis of flap choice and its implications for wound and bone healing, infection control, and restoration of the local microenvironment in complex tissue defects.

## 2 Materials and methods

### 2.1 Experimental model: study overview

**Figure 1:**
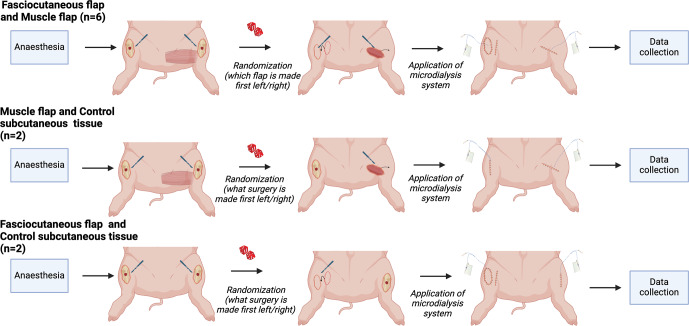
Experimental design. The hindlimbs of the pigs were operated on with a bilateral flap (fasciocutaneous and muscle flaps, n= 6), a muscle flap and control subcutaneous tissue ( n= 2), and a fasciocutaneous flap and control subcutaneous tissue ( n= 2). Created with BioRender.

A total of 10 female Danish Landrace pigs (mean weight: 61 kg; range: 53–69 kg) were included. Using both hindlimbs, symmetric tibial bone and soft-tissue defects were created to imitate a post-surgical or traumatic tissue loss. The defect was reconstructed with either a muscle flap ( n= 8) or a fasciocutaneous flap ( n= 8), or it was closed primarily ( n= 4, controls) (Fig. 1). Randomization determined whether the flap or primary wound closure was performed first. Two pilot animals were studied first to ensure the model's technical feasibility.

**Figure 2:**
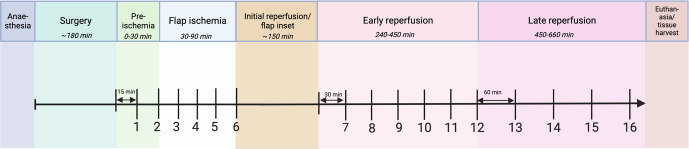
Experimental timeline. Following anaesthesia and surgery, microdialysis samples were collected during “pre-ischemia” (15 min collection), “flap ischemia” (15 min collection), “early reperfusion” (30 min collection), and “late reperfusion” (60 min collection), ending with euthanasia and tissue harvest. Sample numbers are demonstrated below the timeline. Created with BioRender.

Following flap elevation, microdialysis catheters were placed into the flap interstitial space or, for the controls, in the subcutaneous tissue directly overlying the bone defect. To imitate a free tissue transfer, all flaps were subjected to global ischemia for 60 min by placing a microvascular clamp on the vascular pedicle, followed by clamp removal and a reperfusion period of 420 min (Fig. 2). Flap ischemia and reperfusion were confirmed by the cessation and return of blood flow with a Doppler flow probe (Huntleigh Healthcare Ltd, Cardiff, UK). We chose to omit microsurgical anastomosis from the model for simplicity and to avoid anastomotic-related complications and longer surgical time. Clamping of pedicles to simulate free tissue transfer has previously been reported (Sarcon et al., 2025; Krag et al., 2018). Samples of flap and bone tissues for histopathology were collected immediately post-euthanasia.

### 2.2 Surgical procedure

In a supine position, the proximal tibial anteromedial bone surface was exposed. A 4 × 6 cm^2^ soft-tissue defect was created; 1 cm distal to the tibial tuberosity, a 1.5 × 3 cm sheath of periosteum was removed. In the centre, a circular 1 cm diameter bone defect was created, breaching into cancellous bone using a bone burr and curved chisel (Fig. S1 in the Supplement).

Using a rounded periosteal dissector and a 2 mm incision in each corner, a truncated pyramid of periosteum was carefully lifted off the stripped bone. The periosteal truncated pyramid was designed to “anchor” the soft-tissue flaps and to provide a controlled bone–flap interface free of other tissues.

**Figure 3:**
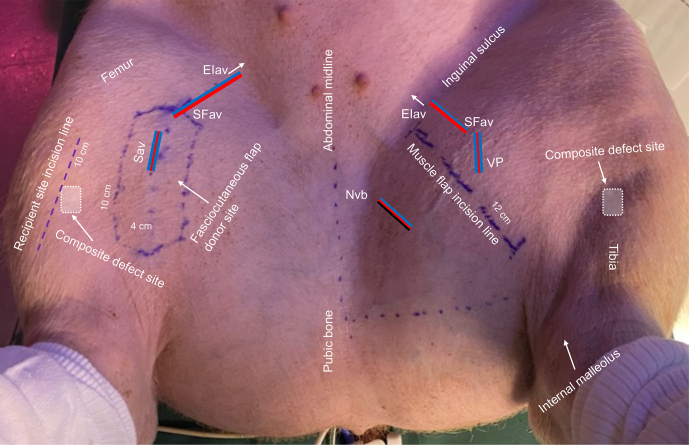
Overview of the porcine model. EIav: external iliac artery and vein origin (arrow); SFav: superficial femoral artery and vein; Sav: saphenous artery and comitant veins; NVb: neurovascular bundle; VP: vascular pedicle. Key anatomical landmarks, incision lines, and measurements are shown.

The gracilis muscle was exposed by a 12 cm incision (Figs. 3 and S2) (Kerrigan et al., 1986; Gonzalez-Garcia et al., 2020). The muscle aponeurosis on the upper tibia was identified laterally and distally and separated from the adjacent saphenous vessels, permitting gracilis' release from the underlying adductor and semimembranosus muscles. Dissection progressed medially and proximally, exposing the deep muscle surface, revealing the neurovascular bundle with the obturator nerve and a minor vascular pedicle medially, as well as the dominant vascular pedicle from the external iliac artery and vein proximally in the inguinal area (Fig. S6). This neurovascular bundle was clamped and transected, isolating the flap on its dominant vascular pedicle. Muscle tissue medial and distal to the neurovascular bundle showed consistent signs of venous congestion and was removed. The flap was transferred as an island flap onto the tibial bone defect and secured in place as the “roof” of the periosteal truncated pyramid by interrupted 4-0 nylon sutures (Fig. S2).

Elevation of the fasciocutaneous flap (consisting of skin, subcutaneous tissue, and fascia) (Kerrigan et al., 1986; Goudot et al., 2022) began by outlining the saphenous vessels from the proximal origin of the external iliac artery and vein using a Doppler flow probe (Figs. 3 and S3) (Goes et al., 2021). The flap measured 4 × 10 cm. An incision was made at the distal flap tip, reaching to the subfascial level, superficial to the gracilis muscle, revealing the vascular pedicle. Distally, the pedicle was clipped and transected (Figs. S3 and S7). The flap was raised from distal to proximal with further dissection of the vascular pedicle proximally until the arc of rotation of the flap was adequate to reach the proximal tibial bone defect. The flap was transferred as an island flap onto the tibial bone defect, maintaining a skin bridge between the flap recipient and donor site (Fig. S3). The flap was secured in place as the muscle flap. Both flaps were elevated from areas devoid of the panniculus carnosus (Rose et al., 1977).

For the limbs selected as controls, primary wound closure was performed by closing the skin directly over the bone and soft-tissue defect. The controls and flap donor sites were sutured with interrupted 3-0 nylon sutures.

During both surgery and the sampling period, the pigs were kept under general anaesthesia with continuous infusion of propofol (10 mg mL^−1^: B. Braun Medical, Frederiksberg, Denmark) and fentanyl (50 µg mL^−1^: Hameln Pharma GmbH, Hameln, Germany). In all pigs, mean arterial pressure was kept at ≥ 65 mmHg. At the end of the experiment, euthanasia was performed with intravenous pentobarbital (Alfasan Nederland BV, the Netherlands).

### 2.3 Microdialysis

Microdialysis is a catheter-based technique used in pre-clinical research and clinically for post-operative flap monitoring by sampling of molecules from the interstitial tissues (Ungerstedt, 1991). Microdialysis catheters (CMA 70, membrane length 30 mm) were placed centrally into the flaps, parallel to the flap axis, using a guide cannula. The catheters were connected to precision pumps (CMA 107) delivering 0.9 % NaCl solution at a rate of 1 µL min^−1^, and samples were instantly stored at - 80 °C. All equipment was acquired from M Dialysis AB (Stockholm, Sweden).

Flap vitality and the microenvironment were assessed based on markers of tissue metabolism (glucose, lactate, and pyruvate), quantified using a CMA 600 MD Analyzer (Sorotos et al., 2024; Rauff-Mortensen et al., 2020). Ischemia and reduced oxygen induce cellular anaerobic metabolism, leading to an increase in the lactate-to-pyruvate ( L/P ) ratio and a reduction in the glucose-to-lactate ( G/L ) ratio. For an applied reference, ischemia was defined as dialysate values of glucose of ≤ 0.2 mmol L^−1^ or values of lactate of ≥ 10 mmol L^−1^ or as an L/P ratio of ≥ 50 or a G/L ratio of ≤ 0.15 (Sorotos et al., 2024; Rauff-Mortensen et al., 2020).

Microdialysis samples were collected in four time periods, with a total of 16 samples (Fig. 2).

### 2.4 Histopathology

Fasciocutaneous and muscle flaps were harvested as a whole and divided into proximal, middle, and distal parts (Fig. S4). The tissues were formalin-fixed (10 % buffered formalin) for approximately 3 weeks, cross-sectionally trimmed, graded-alcohol- and xylene-processed, paraffin-embedded, and stained with haematoxylin and eosin (HE) for pathomorphological evaluation.

Acute inflammatory parameters (neutrophil infiltration, oedema, and hyperaemia) were semi-quantitatively scored (0: absent; 1: minor; 2: moderate; 3: severe) (Sigmund et al., 2021; Morgenstern et al., 2018). For muscle tissue, myofiber necrosis was also noted. All histopathologic assessments and scores were performed and obtained by a trained veterinary pathologist and evaluated by means of standardized histopathological scoring charts (Table S5).

**Table 1:** Summary of selected samples of interstitial metabolites for fasciocutaneous flaps ( n= 8), muscle flaps ( n= 8), and controls ( n= 4) during the defined time periods.

Interstitial	Pre-ischemia	Flap ischemia	Early reperfusion	Late reperfusion
metabolites	(0–30 min)	(30–90 min)	(240–450 min)	(450–660 min)
Glucose (mmol L^−1^)				
Fasciocutaneous flap	3.6 (2.7: 4.5)	1.4 ( - 0.1: 2.8)^a,b^	1.7 (0.5: 2.9)	1.0 (0.1: 1.8)
Muscle flap	3.3 (2.2: 4.4)	1.3 (1.0: 2.7)^b^	2.6 (1.8: 2.5)^a^	1.1 (0.3: 2.0)
Subcutaneous control tissue	5.5 (3.8: 7.1)	3.8 (2.5: 5.1)	2.0 ( - 0.3: 4.2)	2.1 (0.5: 3.7)
Lactate (mmol L^−1^)				
Fasciocutaneous flap	3.4 (2.8: 4.0)	7.8 (6.0: 9.7)^a^	5.7 (4.2: 7.2)^b^	5.9 (5.1: 6.7)^c^
Muscle flap	4.4 (1.4: 7.5)	7.2 (4.8: 9,7)^a^	3.6 (1.8: 5.3)^b^	4.4 (3.0: 5.8)
Control subcutaneous tissue	2.8 (2.6: 3.1)	2.7 (1.7: 3.7)	4.6 (1.4: 7.8)	3.5 (1.7: 5.2)
Pyruvate ( µmol L^−1^)				
Fasciocutaneous flap	151 (131: 171)^b^	101 (83: 118)^b^	162 (132: 192)	101 (82: 119)^b^
Muscle flap	95 (63: 126)^a,b^	28 (18: 38)^a,b^	242 (166: 318)	207 (173: 241)^a,b^
Control subcutaneous tissue	166 (135: 196)	135 (105: 165)	144 (89: 198)	98 (80: 115)
L/P ratio				
Fasciocutaneous flap	23 (19: 26)^b^	87 (49: 124)^a,b^	36 (27: 44)^a,b^	62 (49: 76)^b^
Muscle flap	50 (19: 81)^a,b^	292 (189: 396)^a,b^	14 (11: 17)^a,b^	20 (15: 25)^a,b^
Control subcutaneous tissue	17 (12: 22)	21 (13: 28)	34 (16: 52)	38 (14: 62)
G/L ratio				
Fasciocutaneous flap	1.1 (0.8: 1.4)^a^	0.2 ( - 0.0: 0.5)^a^	0.5 (0.0: 0.9)^b^	0.2 (0.0: 0.4)
Muscle flap	1.1 (0.6: 1.6)	0.2 (0.1: 0.3)	1.1 (0.5: 1.7)^a,b^	0.6 ( - 0.2: 1.4)
Control subcutaneous tissue	1.9 (1.5: 2.3)	1.7 (0.5: 3.0)	0.9 ( - 0.2: 2.0)	0.9 (0.1: 1.8)

Selected samples from different periods are presented as means and the 95 % confidence interval (CI): pre-ischemia (sample no. 2), ischemia (sample no. 6), early Reperfusion (sample no. 7), and late reperfusion (sample no. 16). ^a^ p≤ 0.05 compared with control tissue. ^b^ p≤ 0.05 between-flap comparison. ^c^ p= 0.060 compared with muscle flap.

Bone lesions, located under the flaps, were harvested as a whole (proximal) tibia block from three randomly selected pigs. Bone was formalin-fixed as the soft tissues, decalcified (17 % formic acid) for approximately 6 weeks, processed, and stained with HE as described above.

### 2.5 Statistical analysis

Interstitial metabolite concentrations were calculated as means and L/P and G/L ratios ± standard deviations (Excel v.16.0, Microsoft, NY, United States of America). Metabolite dialysate samples were attributed to selected time points of the sampling interval (Fig. 2). The three groups (fasciocutaneous flap, muscle flap, and control subcutaneous tissue) were compared on a log-transformed scale overall in a repeated-measure mixed-model ANOVA, followed by pairwise comparisons, including the differences between the groups and time periods, using linear regression (Stata/SE v. 18.0, Stata, TX, United States of America). The Kenward–Roger approximation was applied to adjust degrees of freedom due to the small sample size. Model assumptions were tested by assessing residual and fitted value normality.

**Figure 4:**
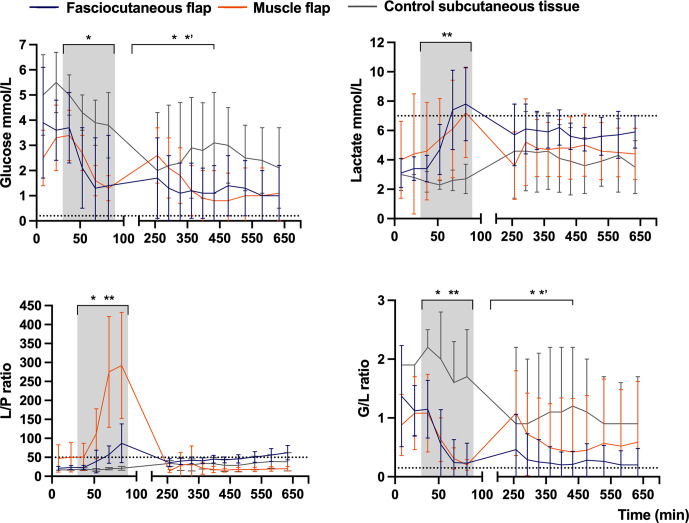
Mean concentration–time curves of glucose, lactate, L/P ratio, and G/L ratio in fasciocutaneous flaps ( n= 8), muscle flaps ( n= 8), and control subcutaneous tissue ( n= 4). Error bars represent the SD. The grey shading marks flap ischemia, and dotted lines mark ischemic cut-off values. The x axis is split for clarity. The p values reflect mixed-model ANOVA comparisons across time periods: (1) pre-ischemia (0–30 min), (2) flap ischemia (30–90 min), (3) early reperfusion (240–450 min), and (4) late reperfusion (450–660 min): ^*^ p≤ 0.05 between flap types, ^**^ p≤ 0.05 between flap types and controls, ${}^{*}{}^{\prime }$ p≤ 0.05 between muscle flap and controls. Values are based on raw data.

The fasciocutaneous and muscle flap histopathological scores were compared (GraphPad Prism in v. 10.2.2, CA, United States of America) using one-way ANOVA with Tukey's multiple comparisons of the average scores of the (1) epidermis, dermis, subcutaneous tissue, and fascia and the (2) epimysium, perimysium, and endomysium across the proximal, middle, and distal parts of the flaps.

The significance level was set at p≤ 0.05. No power analysis was done, although similar studies have indicated that a sample size of six to eight pigs per group is sufficient to detect significant effects (Rauff-Mortensen et al., 2020; Krag et al., 2017, 2018).

## 3 Results

All pigs ( n= 10) and flaps completed the study. Flap viability was confirmed macroscopically. Pre-ischemic dialysate samples from two pigs (fasciocutaneous flap n= 1, muscle flap n= 1, and controls n= 2) were not collected due to initial catheter malfunction, and all metabolites from one muscle flap were excluded due to an analytical error. All flaps underwent histopathological analyses.

### 3.1 Metabolic changes: ischemia

In the fasciocutaneous and muscle flaps, ischemia caused significant metabolic changes compared to control tissue, with (1) rapid decreases in glucose concentrations and the G/L ratio and (2) increases in the lactate concentration and the L/P ratio (Table 1 and Fig. 4). Between-flap comparisons revealed no differences in the magnitude of changes across investigated markers, except for the L/P ratio, which increased 3-fold in the muscle flaps compared to the fasciocutaneous flaps. In both flap types, the lactate concentration and the L/P ratio exceeded the ischemic cut-off values. The glucose concentration and the G/L ratio did not decrease below the ischemic cut-off values in either flap type, although the G/L ratio approached the threshold in both flap types. In the non-ischemic control tissue, no metabolite marker surpassed any threshold. Between-flap comparisons demonstrated the promptest acquisition of ischemic cut-off values for the lactate concentrations in the fasciocutaneous flap and for the L/P ratio in the muscle flap.

### 3.2 Metabolic changes: reperfusion

In the fasciocutaneous and muscle flaps, reperfusion caused a significant metabolic reversal compared to control tissue, with (1) rapid increases in glucose concentrations and the G/L ratio and (2) reductions in lactate concentrations and L/P ratios (Table 1 and Fig. 4). Between-flap comparisons demonstrated that lactate and L/P ratio levels remained 1.5-fold higher during the reperfusion period in the fasciocutaneous flap. Neither the flaps nor the control tissues surpassed the ischemic cut-off values of any investigated marker, except for the L/P ratio (closely approached by the G/L ratio) in the fasciocutaneous flaps during late reperfusion.

**Figure 5:**
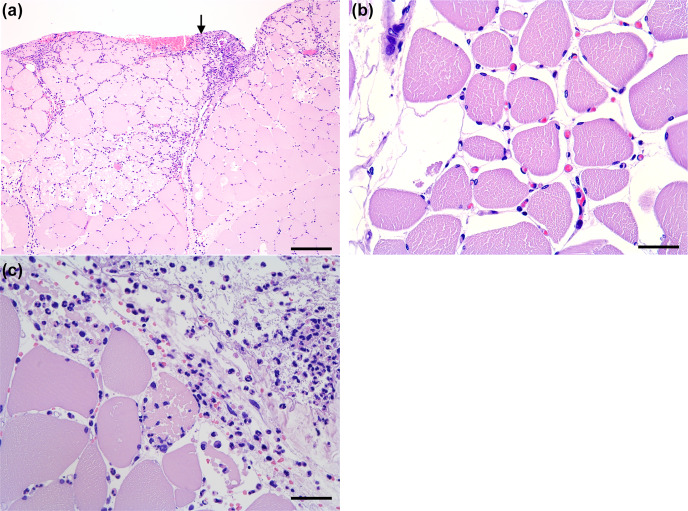
Histology of the muscle flaps (HE-stained). **(A)** Severe neutrophil infiltration in the perimysium and endomysium, with the bar showing 250 µm . The arrow indicates the interface towards the bone lesion. **(B)** Moderate oedema and hyperaemia with erythrocyte-filled capillaries, with the bar showing 50 µm . **(C)** Distal biopsy facing the bone lesion. Occasional myofiber necrosis with severe neutrophil infiltration, with the bar showing 50 µm .

### 3.3 Histopathology

**Figure 6:**
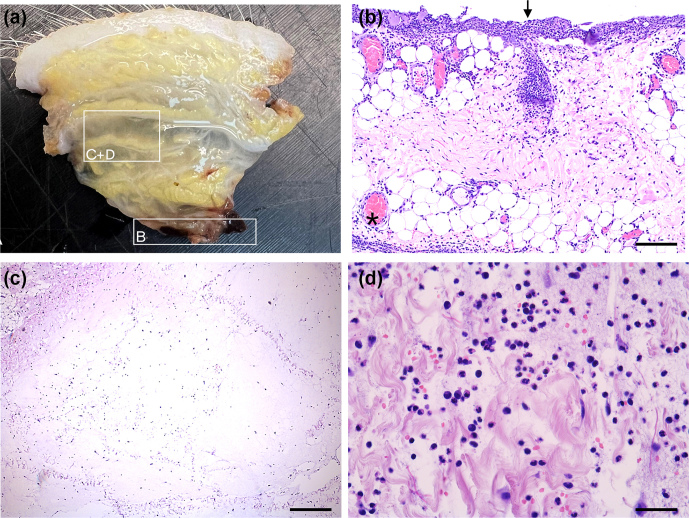
Histology of fasciocutaneous flaps (HE-stained). **(A)** Flap before embedding displaying oedema. **(B)** Severe neutrophil infiltration at bone–flap interface (arrow). Severe hyperaemia in subcutaneous vessels (asterisk), with the bar showing 200 µm . **(C)** Severe subcutaneous oedema with enlarged interstitial spaces and neutrophils, with the bar showing 400 µm . **(D)** Higher magnification of severe subcutaneous oedema and neutrophils, with the bar showing 50 µm .

**Figure 7:**
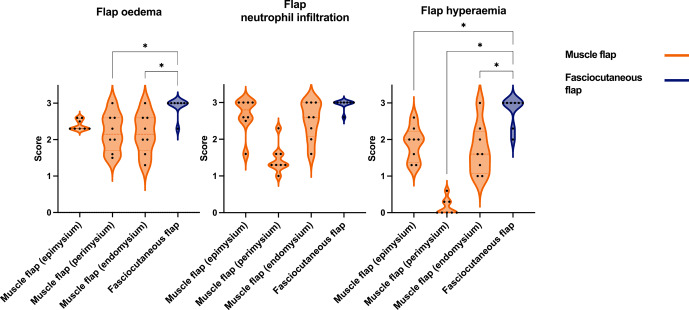
Comparison of acute inflammatory scores (flap oedema, flap neutrophil infiltration, flap hyperaemia) in muscle and fasciocutaneous flaps. Muscle flaps (orange) showed variable scores in the epimysium, perimysium, and endomysium. Fasciocutaneous flaps (blue) showed moderate to severe scores for all parameters in the subcutaneous tissue and fascia, while the epidermis showed no response (not shown). The p values are obtained by means of one-way ANOVA with Tukey's multiple comparisons. ^*^ p≤ 0.05 (non-significant comparisons are not shown).

All flaps showed acute inflammatory changes (Figs. 5 and 6), dominated by neutrophil infiltration, oedema, and hyperaemia with consistent scores across locations (proximal, middle, and distal parts). Overall, fasciocutaneous flaps demonstrated significantly higher mean inflammatory scores than muscle flaps, except for neutrophil infiltration (Fig. 7). In both flap types, neutrophil infiltration was most pronounced at the bone–flap interface, with moderate hyperaemia also present in this area for muscle flaps. Sporadic myofiber necrosis was observed in all flaps. All cortical bone lesions expanded into cancellous bone with adjacent marrow oedema. No cellular inflammatory response was observed in the bone.

## 4 Discussion

This is, to our knowledge, the first porcine model developed to characterize how muscle and fasciocutaneous flaps influence the microenvironment at the bone lesion–flap interface. The model enabled combined functional assessments using microdialysis and structural comparisons through histopathology. The main finding was the establishment of a reproducible comparative model demonstrating consistent and distinct early responses between flap types across interstitial metabolites and histopathology.

In both flaps, ischemia caused rapid decreases in glucose and G/L ratios and increases in lactate and L/P ratios, followed by reversed changes during reperfusion. These findings align with clinical and experimental microdialysis reports on metabolite fluctuations during flap ischemia–reperfusion (Röjdmark et al., 2002, 2000; Setala et al., 2006, 2004; Sorotos et al., 2024; Rauff-Mortensen et al., 2020). This shift reflects the transition from aerobic to anaerobic metabolism due to blood supply disruption. The between-flap differences in L/P and G/L ratios and the slower glucose recovery in fasciocutaneous flaps during early reperfusion are consistent with previous studies (Röjdmark et al., 2002; Setala et al., 2004). These distinct patterns likely stem from tissue-specific metabolism: muscle tissue has a high metabolic rate with many mitochondria and a mixed energy source profile, whereas subcutaneous tissue relies more on fatty acids (Röjdmark et al., 2002; Hanberg et al., 2021; Sorotos et al., 2024). Such differences may alter the microenvironment beneath the flaps, with implications for tissue healing and interaction with bacterial contamination and colonization (Bjarnsholt et al., 2022).

In parallel with the metabolic alterations, flap morphology revealed pronounced inflammatory responses. Flap elevation and ischemia–reperfusion injury are recognized inducers of acute cellular and vascular inflammation (Heden et al., 1989; Heden and Sollevi, 1989). We observed consistent neutrophil infiltration, oedema, and hyperaemia throughout both flap types, with significantly higher mean scores for oedema and hyperaemia in the fasciocutaneous flaps. At the bone lesion–flap interface, both flaps showed increased neutrophil infiltration, while the muscle flaps displayed locally enhanced hyperaemia. This early cellular activity and local active hyperaemia at the interface suggest the onset of immune-mediated remodelling and biological interaction with bone. Such interface activity aligns with reports of enhanced muscle-flap-dependent blood flow and increased ingrowth beneath muscle flaps (Gosain et al., 1990; Calderon et al., 1986). In the fasciocutaneous flaps, hyperaemia was widespread, accompanied by co-existing oedema and, thus, is more likely to be a result of impaired microvascular venous return, i.e. passive hyperaemia (Hjortdal et al., 1992; Heden and Sollevi, 1989). Together, these findings support the presence of multiple mechanisms including the “no-reflow or slow-reflow” hypoperfusion phenomena following ischemia–reperfusion (May et al., 1978; Rezkalla and Kloner, 2002). Despite the detailed pathophysiology of flap ischemia–reperfusion being beyond the scope of the present study, the resultant structural changes and early cellular responses at the bone lesion–flap interface merit emphasis.

The observed inflammatory changes were accompanied by marked oedema, reflecting altered hydrostatic and colloid osmotic pressures. Although such perfusion changes typically resolve within days, they may critically affect early flap function as a supplier of, for example, oxygen, nutrients, immune cells, growth factors, and systemically administered antibiotics (May et al., 1978; Gosain et al., 1990). Impaired flap function could compromise the tissue-healing microenvironment and infection control during this vulnerable period (Bjarnsholt et al., 2022). The higher inflammatory scores observed in the fasciocutaneous flaps suggest microvascular and structural differences that may explain the elevated lactate and L/P ratio during late reperfusion. In the acute setting, these findings could indicate superior early healing and infection control potential of muscle flaps. However, long-term studies are required for a comprehensive comparison, including assessments of perfusion as a surrogate for wound healing, antibiotic delivery, and bacterial clearance.

Several limitations should be acknowledged. This study was conducted in non-infected pigs as part of a broader protocol investigating other interstitial parameters and sites, and no microsurgical anastomoses were performed. Consequently, micro-embolisms from anastomoses were not represented (Acland et al., 1989). During flap inset and placement of microdialysis catheters in other compartments, sampling was paused for a mean of 150 min between ischemia and early reperfusion. Regarding microdialysis technicalities, metabolite concentrations are relative as no calibration or full equilibrium was achieved (Ungerstedt, 1991; Rosdahl et al., 1998). The between-flap and flap control comparisons were performed within the same animal, without muscular control tissue. This design reduced animal use and inter-individual variability but may have introduced confounding from the sequential nature of the surgical interventions and the limited sample size. Nevertheless, repeated metabolite sampling, spatial histopathological analyses, mixed-model statistics, and randomization of the reconstruction order were incorporated to strengthen the robustness of these findings.

## 5 Conclusions

In conclusion, we established a reproducible comparative large-animal model combining microdialysis and histopathology to characterize early flap-mediated tissue responses. Following brief ischemia–reperfusion, fasciocutaneous flaps exhibited more pronounced structural alterations and slower metabolic recovery compared with muscle flaps. These distinct early responses may influence subsequent tissue healing and infection control. Longer-term studies are warranted to assess flap remodelling, bone regeneration, bacterial clearance, and antibiotic delivery. This model provides a translational platform for advancing our understanding of flap-supported healing microenvironments and for guiding ortho-plastic reconstructive strategies.

## Supplement

10.5194/jbji-10-597-2025-supplement The supplement related to this article is available online at https://doi.org/10.5194/jbji-10-597-2025-supplement.

## Data Availability

Data will be made available upon reasonable request.
