# Supplementary material for: A human-scale porcine fasciocutaneous and muscle flap model for the evaluation of ortho-plastic reconstructions of lower-limb defects
Source: J Bone Jt Infect. 2025 Dec 10;10(6):597–607. doi: 10.5194/jbji-10-597-2025 (PMC12753138; doi:10.5194/jbji-10-597-2025)
Supplement: The supplement related to this article is available online at https://doi.org/10.5194/jbji-10-597-2025-supplement. [file jbji-10-597-2025-supplement.pdf]

Supplement of J. Bone Joint Infect., 10, 597–607, 2025
https://doi.org/10.5194/jbji-10-597-2025-supplement
© Author(s) 2025. CC BY 4.0 License.

Supplement of

A human-scale porcine fasciocutaneous and muscle flap model for the evaluation of ortho-plastic reconstructions of lower-limb defects

Josefine Slater et al.

Correspondence to: Josefine Slater (josefine.slater@clin.au.dk)

The copyright of individual parts of the supplement might differ from the article licence.

Supplementary

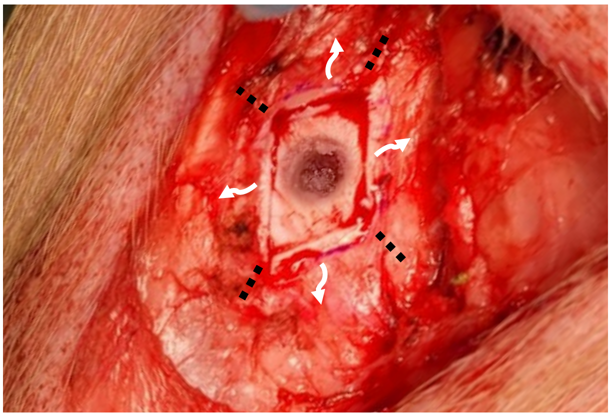

Supplementary Figure S1: The truncated periosteal pyramid and central bone defect breaching into cancellous bone. The periosteal truncated pyramid was designed to "anchor" the soft tissue flaps, and to provide a controlled bone-flap interface free of other tissues. The black perpendicular dotted lines demonstrate 2-mm incision lines, and the white arrows show the direction in which the periosteum was carefully lifted of the stripped bone.

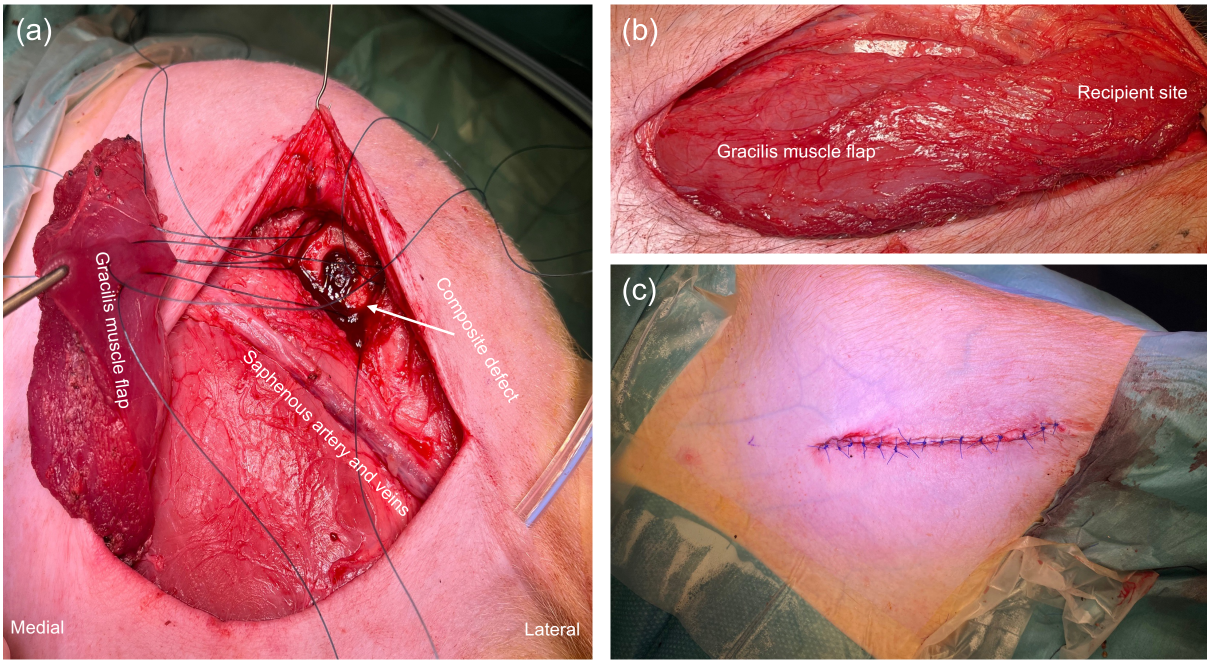

Supplementary Figure S2: A and B) The left hindlimb and gracilis muscle flap with the recipient site. The composite defect consisting of bone, periosteum, and soft tissues is also depicted with the sutures in place. The saphenous artery and veins marking the lateral boarder of the flap are demonstrated. C) The skin was closed with simple interrupted sutures following microdialysis catheter placement (not shown).

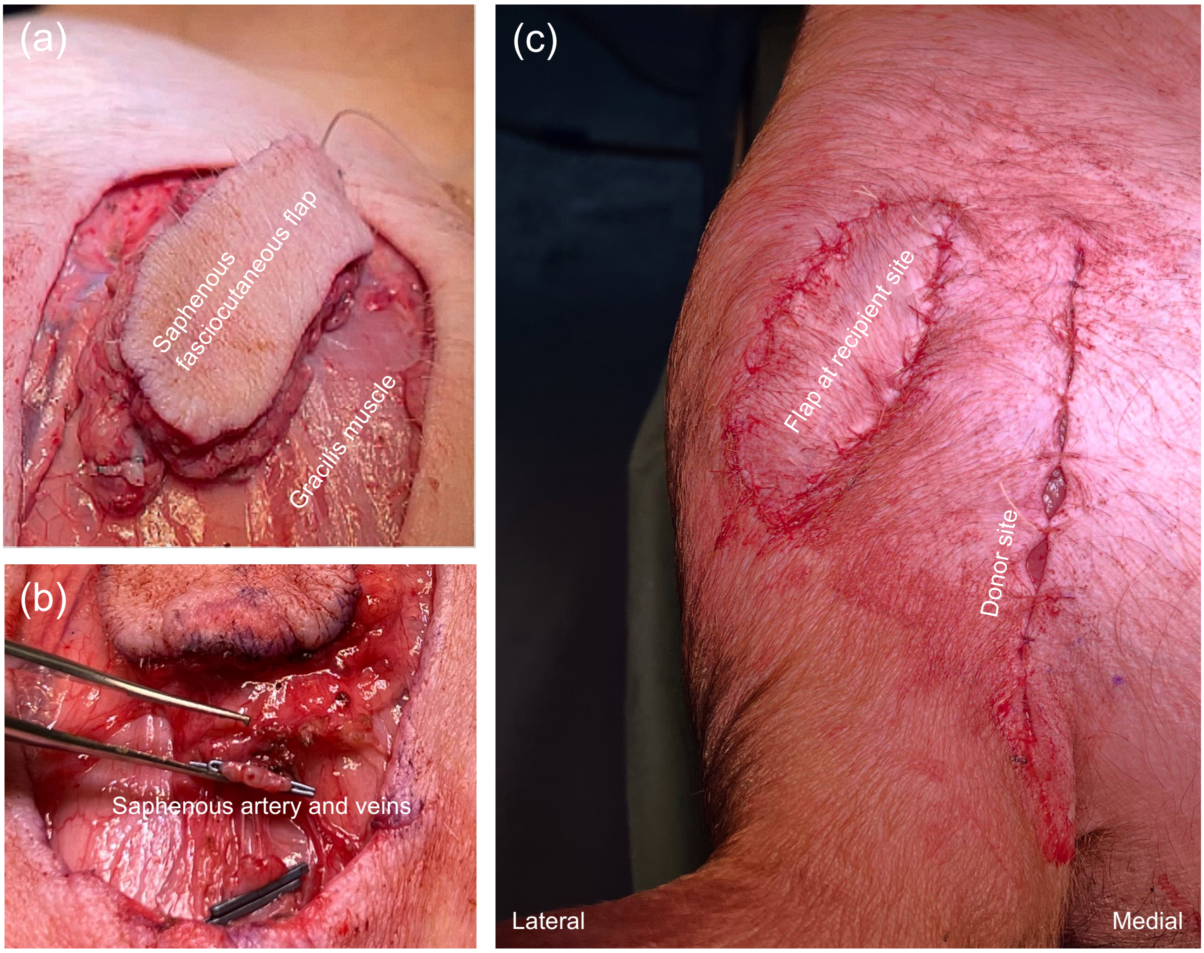

Supplementary Figure S3: A and C) The right hindlimb and saphenous fasciocutaneous flap raised as an island flap based on the saphenous artery and veins, with the donor site corresponding to the original skin paddle harvest area. The recipient site with the flap in place is also depicted. B) The ligated saphenous artery and veins.

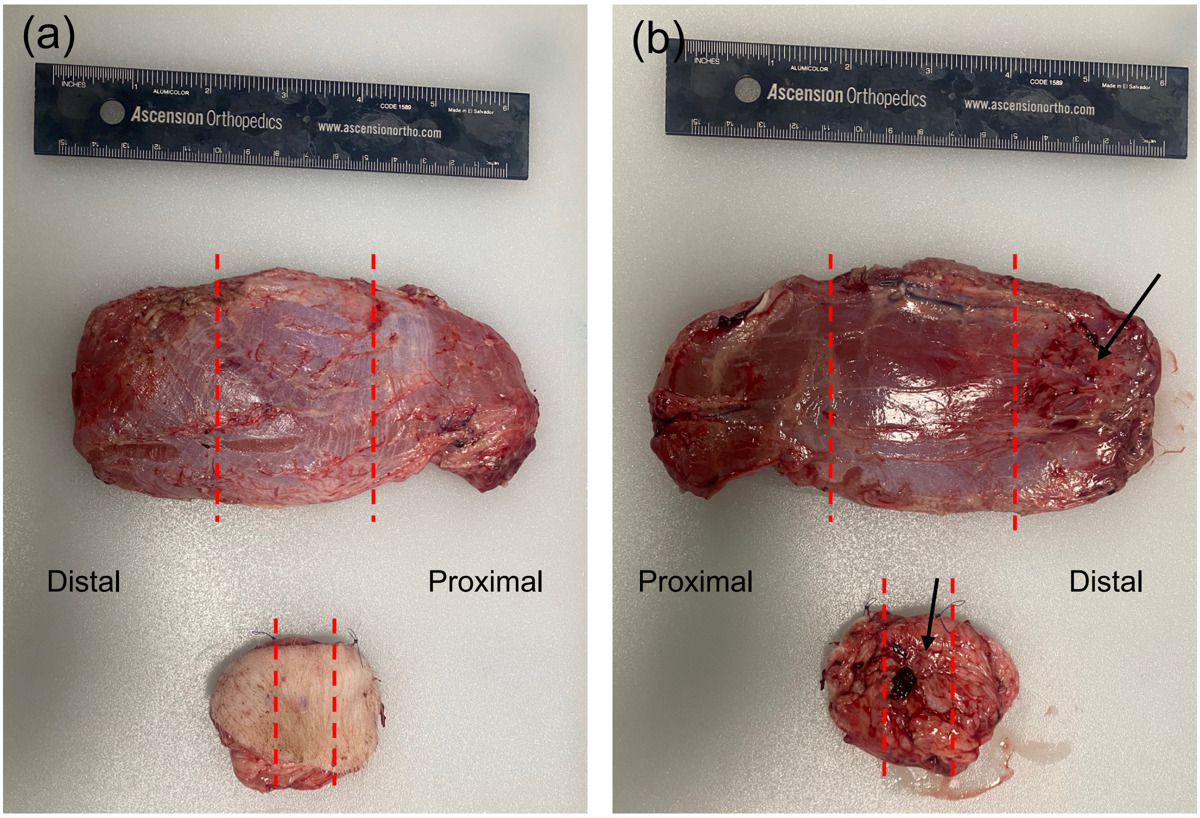

Supplementary Figure S4: The flaps immediately after harvest (A and B). A) demonstrates the superficial surface, and B) the deep surface. The red lines demonstrate the divisions into proximal, middle, and distal parts. The arrow demonstrates the area of the flap that was in direct relation to the bone defect. Following the tissues were fixated in 10% buffered formalin for approximately three weeks, cross-sectionally trimmed, processed through graded alcohol and xylene, and embedded in paraffin wax. Sections (2-3 mm) were cut and stained with Haematoxylin and Eosin (HE) for pathomorphological evaluation.

Animal ID	Muscle flap location	Epimysium			Perimysium			Endomysium		
		Oedema	Hyperaemia	Neutrophil infiltration	Oedema	Hyperaemia	Neutrophil infiltration	Oedema	Hyperaemia	Neutrophil infiltration
	Proximal	*0,1,2,3*	*0,1,2,3*	*0,1,2,3*	*0,1,2,3*	*0,1,2,3*	*0,1,2,3*	*0,1,2,3*	*0,1,2,3*	*0,1,2,3*
	Middle	*0,1,2,3*	*0,1,2,3*	*0,1,2,3*	*0,1,2,3*	*0,1,2,3*	*0,1,2,3*	*0,1,2,3*	*0,1,2,3*	*0,1,2,3*
	Distal	*0,1,2,3*	*0,1,2,3*	*0,1,2,3*	*0,1,2,3*	*0,1,2,3*	*0,1,2,3*	*0,1,2,3*	*0,1,2,3*	*0,1,2,3*
	Average									

Animal ID	Fascio-cutaneous flap location	Epidermis			Dermis			Subcutis + fascia		
		Oedema	Hyperaemia	Neutrophil infiltration	Oedema	Hyperaemia	Neutrophil infiltration	Oedema	Hyperaemia	Neutrophil infiltration
	Proximal	*0,1,2,3*	*0,1,2,3*	*0,1,2,3*	*0,1,2,3*	*0,1,2,3*	*0,1,2,3*	*0,1,2,3*	*0,1,2,3*	*0,1,2,3*
	Middle	*0,1,2,3*	*0,1,2,3*	*0,1,2,3*	*0,1,2,3*	*0,1,2,3*	*0,1,2,3*	*0,1,2,3*	*0,1,2,3*	*0,1,2,3*
	Distal	*0,1,2,3*	*0,1,2,3*	*0,1,2,3*	*0,1,2,3*	*0,1,2,3*	*0,1,2,3*	*0,1,2,3*	*0,1,2,3*	*0,1,2,3*
	Average									

Supplementary Table S5: The pathohistological variables were graded separately for each animal according to location with the following scoring chart; 0: absent, 1: minor, 2: moderate, 3: severe. Neutrophil infiltration was defined as neutrophil granulocytes outside vessels, oedema as enlarged interstitial spaces, and hyperaemia as dilated vessels with flat elongated endothelial cells filled with erythrocytes. The flap slides were scored separately, i.e., 1) epidermis, dermis, subcutis, and fascia, and 2) epimysium, perimysium, and endomysium, respectively.

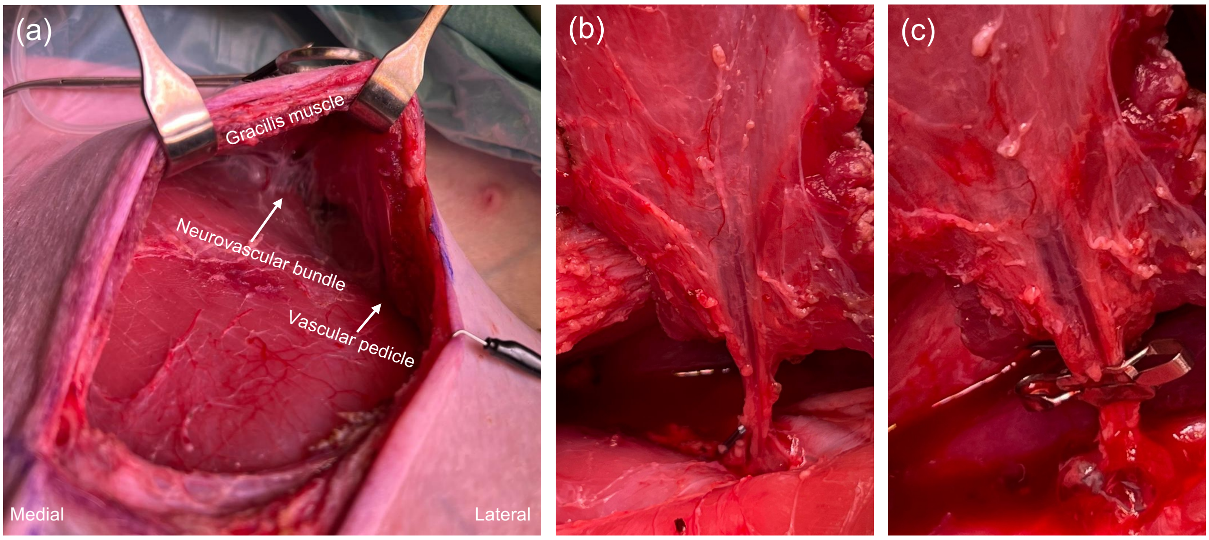

Supplementary Figure S6: A) The gracilis muscle is elevated demonstrating the neurovascular bundle (arrow) and vascular pedicle (arrow), and underlying semimembranosus muscle. B and C) The vascular pedicle with the artery and veins without and with microvascular clamps, respectively.

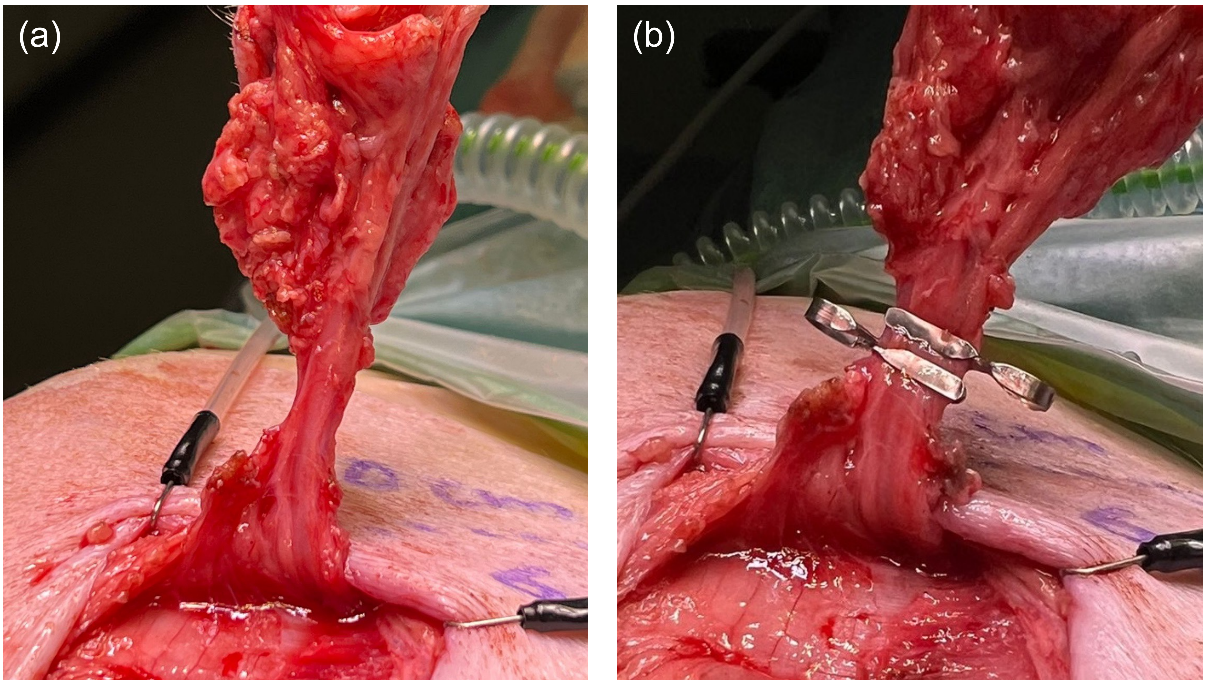

Supplementary Figure S7: A and B) The saphenous fasciocutaneous flap with the vascular pedicle consisting of the saphenous artery and veins, without and with microvascular clamps, respectively.
